# Comparison of Comprehensive Serum miRNA Sequencing and Apolipoprotein A2 Isoforms for Early Detection of Pancreatic Cancer

**DOI:** 10.3390/cancers18071177

**Published:** 2026-04-07

**Authors:** Hirotaka Kashima, Munenori Kawai, Kei Iimori, Munemasa Nagao, Takamitsu J. Morikawa, Ryo Otomo, Mitsuharu Hirai, Kosuke Minaga, Masanori Asada, Atsushi Umemura, Yoshito Uenoyama, Toshihiro Morita, Shujiro Yazumi, Ryuki Minami, Saiko Marui, Yuki Yamauchi, Yoshitaka Nakai, Yutaka Takada, Seiji Shio, Takuto Yoshioka, Naoki Kanda, Tomonori Masuda, Kazuyuki Nagai, Etsuro Hatano, Akihisa Fukuda, Hiroshi Seno

**Affiliations:** 1 Department of Gastroenterology and Hepatology, Kyoto University Graduate School of Medicine, 54 Kawahara-cho, Shogoin, Sakyo-ku, Kyoto 606-8507, Japan; hirotakak@kuhp.kyoto-u.ac.jp (H.K.); mkawai@kuhp.kyoto-u.ac.jp (M.K.); kei_iimori@kuhp.kyoto-u.ac.jp (K.I.); ds110559@kuhp.kyoto-u.ac.jp (M.N.);; 2 Research and Development Division, ARKRAY, Inc., Yousuien-nai, 59 Gansuin-cho, Kamigyo-ku, Kyoto 606-8507, Japan; 3 Department of Gastroenterology and Hepatology, Kindai University Faculty of Medicine, Osaka 589-8511, Japan; 4 Department of Gastroenterology and Hepatology, Japanese Red Cross Osaka Hospital, Osaka 543-8555, Japan; m-asada@osaka-med.jrc.or.jp; 5 Department of Pharmacology, Kyoto Prefectural University of Medicine, Kyoto 602-8566, Japan; aumemura@koto.kpu-m.ac.jp; 6 Department of Gastroenterology and Hepatology, Japanese Red Cross Wakayama Medical Center, Wakayama 640-8558, Japan; 7 Department of Gastroenterology and Hepatology, Kitano Hospital, Tazuke Kofukai Medical Research Institute, Osaka 530-8480, Japans-yazumi@kitano-hp.or.jp (S.Y.); 8 Department of Gastroenterology, Tenri Hospital, Nara 632-0032, Japan; 9 Department of Gastroenterology and Hepatology, Shiga General Hospital, Moriyama 524-8524, Japan; 10 Department of Gastroenterology, Hyogo Prefectural Amagasaki General Medical Center, Amagasaki 661-0892, Japan; yyuki@kuhp.kyoto-u.ac.jp; 11 Department of Gastroenterology and Hepatology, Kyoto Katsura Hospital, Kyoto 615-8256, Japan; yoshi-nakai@katsura.com; 12 Department of Gastroenterology and Hepatology, Kobe City Nishi-Kobe Medical Center, Kobe 651-2273, Japan; yutakada@kuhp.kyoto-u.ac.jp; 13 Division of Gastroenterology, Shinko Hospital, Kobe 651-0072, Japan; 14 Department of Gastroenterology and Hepatology, Takatsuki Red Cross Hospital, Takatsuki 569-1096, Japan; takuto@kuhp.kyoto-u.ac.jp (T.Y.);; 15 Department of Gastroenterology and Hepatology, Japanese Red Cross Otsu Hospital, Otsu 520-0046, Japan; tomo_masuda@kuhp.kyoto-u.ac.jp; 16 Division of Hepato-Biliary-Pancreatic Surgery and Transplantation, Department of Surgery, Kyoto University Graduate School of Medicine, Kyoto 606-8501, Japan

**Keywords:** pancreatic cancer, miRNA, NGS, APOA2 isoform, CA19-9

## Abstract

Backgrounds and Aim: Pancreatic cancer is frequently diagnosed at advanced stages, highlighting the need for biomarkers that are capable of detecting early-stage disease in asymptomatic individuals. Recently, apolipoprotein A2 isoforms (ApoA2-ATQ/AT) have been reported as a new blood biomarker for pancreatic cancer. We recently developed diagnostic models based on 100 highly expressed serum microRNAs (miRNAs) combined with CA19-9; these models achieved high accuracy in terms of distinguishing individuals with pancreatic cancer from healthy individuals. This study aimed to compare the diagnostic performance of these miRNA-based models with that of the ApoA2-ATQ/AT biomarker. Methods: Comprehensive sequencing of serum miRNAs was conducted using samples from 120 pancreatic cancer patients recruited across 14 hospitals, along with 93 healthy controls without cancer. Serum CA19-9 levels, miRNA index values, miRNA+CA19-9 index values, and ApoA2 index values were assessed. miRNA-based indices were derived from classification models built on an automated machine-learning platform. Results: The miRNA model (AUC 0.94; 95% CI 0.91–0.97) and the miRNA+CA19-9 model (AUC 0.99; 95% CI 0.98–1.00) outperformed ApoA2 (AUC 0.89; 95% CI 0.84–0.93) in terms of distinguishing individuals with pancreatic cancer from healthy controls across all stages. In early-stage disease (stages 0–I and 0–II), both miRNA-based models also demonstrated superior performance. Strong negative correlations were observed between the ApoA2 index and both the miRNA model index (r = −0.62) and the miRNA+CA19-9 index (r = −0.63). Conclusions: These findings suggest that miRNA-based diagnostic models, particularly when combined with CA19-9, could serve as powerful tools for the early detection of pancreatic cancer.

## Simple Summary

Pancreatic cancer is frequently diagnosed at advanced stages, highlighting the need for biomarkers that are capable of detecting early-stage disease in asymptomatic individuals. Recently, apolipoprotein A2 isoforms (ApoA2-ATQ/AT) have been reported as a new blood biomarker for pancreatic cancer. We recently developed diagnostic models based on 100 highly expressed serum microRNAs (miRNAs) combined with CA19-9. These models achieved high accuracy in terms of distinguishing individuals with pancreatic cancer from healthy individuals. This study aimed to compare the diagnostic performance of these miRNA-based models with that of the ApoA2-ATQ/AT biomarker. Comprehensive serum miRNA sequencing was performed on samples from 120 patients with pancreatic cancer, who were collected from 14 hospitals, and 93 non-cancer healthy controls. Using distinction models constructed with an automated machine learning platform, miRNA-based indices were generated. The miRNA model (AUC 0.94) and the miRNA+CA19-9 model (AUC 0.99) showed higher accuracy compared with ApoA2 (AUC 0.89) in terms of distinguishing individuals with pancreatic cancer from healthy controls across all stages, including early-stage disease.

## 1. Introduction

Pancreatic cancer ranks as the fourth leading cause of cancer-related mortality worldwide [1]. It is associated with a very poor prognosis, with a five-year survival rate of only 5–15% [2]. Patients who are eligible for and benefit from surgical resection have comparatively better outcomes; under the Union for International Cancer Control (UICC) staging system, the five-year survival rate for stage 0–IB disease exceeds 60% [3,4]. By contrast, for stages III and IV—where curative surgery is generally not possible—five-year survival rates drop to 2.5% and 0.9%, respectively [4]. These data underscore the importance of detecting pancreatic cancer at a localized, resectable stage to improve survival.

Early stage pancreatic cancer is often asymptomatic, or individuals present with nonspecific symptoms, making the detection of the disease difficult. Consequently, only 12% of cases are diagnosed at stages 0–IB [5], and most cases are locally advanced or have metastasis when cancers are detected [6,7].

At present, carbohydrate antigen 19-9 (CA19-9) is widely used in clinical practice as a serum biomarker for pancreatic cancer. Nevertheless, CA19-9 levels typically increase only in advanced stages, limiting the utility of this biomarker for detecting asymptomatic or early-stage disease [8]. Therefore, there is an urgent need to identify new serum biomarkers that are capable of enabling an early diagnosis of pancreatic cancer.

MicroRNAs (miRNAs) are genome-encoded non-coding RNAs that do not undergo translation after transcription. Instead, they regulate messenger RNA (mRNA) degradation and translational repression by forming complexes and interfering with target mRNAs in the cytoplasm. In this way, they influence biological processes such as development, differentiation, and proliferation [9,10,11,12].

Recently, it has been reported that cancer-associated miRNAs enhance cancer cell migration and invasion and promote tumor metastasis [13]. Moreover, they are stable within exosomes and can be measured in serum, making them attractive candidates for noninvasive cancer biomarkers [14].

It was recently shown by Kashiro et al. that one of the circulating homodimers of apolipoprotein-A2, APOA2-ATQ/AT, is significantly reduced in patients with pancreatic cancer, and that its clinical performance as a blood biomarker is equivalent to or better than that of CA19-9 [15]. On the other hand, we recently showed that a diagnostic model with a combination of 100 highly expressed microRNAs (miRNAs) and CA19-9 could discriminate patients with pancreatic cancer from non-cancer healthy controls with high accuracy [16]. To date, a direct comparison between the diagnostic model that combines the 100 highly expressed miRNAs (as shown in Table S1) with CA19-9 and APOA2-ATQ/AT in terms of their diagnostic accuracy in pancreatic cancer has not been made. Therefore, this study aimed to directly compare the diagnostic accuracy of these two models, especially in the early stages of pancreatic cancer. In this study, the diagnostic accuracy of the miRNAs and CA19-9 model in APOA2-ATQ/AT-negative pancreatic cancer patients was also investigated. 

## 2. Materials and Methods

### 2.1. Patients and Sample Preparation

Serum specimens were collected from 120 patients with pancreatic cancer who were admitted to or referred to 14 secondary/tertiary care hospitals between 2020 and 2023: Kyoto University Hospital (KUHP; *n* = 33), Kindai University Hospital (KDUH; *n* = 40), Kyoto Prefectural University of Medicine (KPUM; *n* = 16), Hyogo Prefectural Amagasaki General Medical Center (AGMC; *n* = 4), Japanese Red Cross Osaka Hospital (JRCOS; *n* = 6), Japanese Red Cross Otsu Hospital (JRCOT; *n* = 4), Japanese Red Cross Takatsuki Hospital (JRCT; *n* = 3), Japanese Red Cross Wakayama Medical Center (JRCW; *n* = 4), Kyoto Katsura Hospital (KKTR; *n* = 1), Kobe City Nishi-Kobe Medical Center (KNMC; *n* = 2), Kitano Hospital (KTNH; *n* = 3), Shiga General Hospital (SGH; *n* = 1), Shinko Hospital (SKHP; *n* = 2), and Tenri Hospital (TNRH; *n* = 1). All samples were stored at −80 °C.

Patients were eligible if they were older than 20 years and had pancreatic cancer confirmed either histologically in a surgically resected specimen or—among those who did not undergo surgery—by CT evidence of a solid pancreatic mass and/or pancreatic duct dilatation together with pancreatic histology or cytology confirming adenocarcinoma. Clinical staging was determined by CT according to the UICC 7th edition criteria.

In addition, 93 serum samples from cancer-free healthy individuals were obtained from three independent cohorts. These cohorts comprised volunteers aged > 40 years recruited in 2021 from the OCROM clinic (OCROMC; *n* = 32), Osaka Pharmacology Clinical Research Hospital (OPHACH; *n* = 28), and ToCROM clinic (TOCROMC; *n* = 33), all primary care institutions; samples were stored at −80 °C. Healthy controls were required to have no history of malignant tumors based on self-reported medical history at the time of blood collection and again one year later. The self-administered questionnaire also inquired about the presence of pancreatic diseases, including chronic pancreatitis, intraductal papillary mucinous neoplasm (IPMN), and pancreaticobiliary maljunction, as well as other diseases; none of the healthy controls reported a history of pancreatic disease.

### 2.2. APOA2 Index Measurement

Serum APOA2 index levels were measured in Showa Medical Science Co., Ltd. using a Toray APOA2-iTQ (Toray, Tokyo, Japan).

### 2.3. CA19-9, miRNA Index, and miRNA+CA19-9 Index

The data of serum CA19-9 levels, serum miRNA index levels, and serum miRNA+CA19-9 index levels were obtained from M. Kawai et al. [16]. In that study, the miRNA model and the miRNA+CA19-9 model were developed using a discovery cohort and independently validated using a validation cohort. The samples used in the present study correspond to the validation cohort, and the models were not re-trained on these samples. Serum CA19-9 concentrations were determined with the Elecsys CA19-9 II assay (Roche Diagnostics Japan, Tokyo, Japan). miRNA libraries were constructed using the QIAseq miRNA Library Kit (QIAGEN K.K., Tokyo, Japan; 331509) together with the QIAseq miRNA NGS 96 Index IL (96) (QIAGEN K.K., Tokyo, Japan; 331565), and were sequenced on a NextSeq 550 system (Illumina K.K., Tokyo, Japan; RRID:SCR_016381) with the NextSeq 500/550 High Output Kit v2.5 (75 cycles) (Illumina K.K., Tokyo, Japan; 20024906). Serum miRNA index values and miRNA+CA19-9 index values were computed using pancreatic cancer classification models developed on the DataRobot AutoML platform (v8.0.12; Boston, MA, USA).

### 2.4. Statistical Analysis

Box plots, scatter plots, receiver-operating characteristic (ROC) curves, and AUC calculations were conducted using the statistical analysis software R (version 4.2.3). To evaluate the performances of APOA2-iTQ, CA19-9, the miRNA model, and the miRNA+CA19-9 model in terms of sensitivity and specificity, 95% confidence intervals (CIs) were calculated using roc.test in the R “pROC” package. The AUC values of the ROC curves with the early-stage pancreatic cancers of the constructed models were calculated using the roc.test in the R “pROC” package.

## 3. Results

### 3.1. Clinical Information

In this study, we analyzed 213 serum samples in total, including 120 from patients with pancreatic cancer and 93 from healthy controls. Participant characteristics are summarized in Table 1. Because recruitment of healthy volunteers was subject to sampling constraints, the pancreatic cancer and control groups differed significantly in age, smoking history, and the prevalence of diabetes mellitus (*p* < 0.05).

### 3.2. Comparison of APOA2, CA19-9, the miRNA Model, and the miRNA+CA19-9 Model

**Figure 1:**
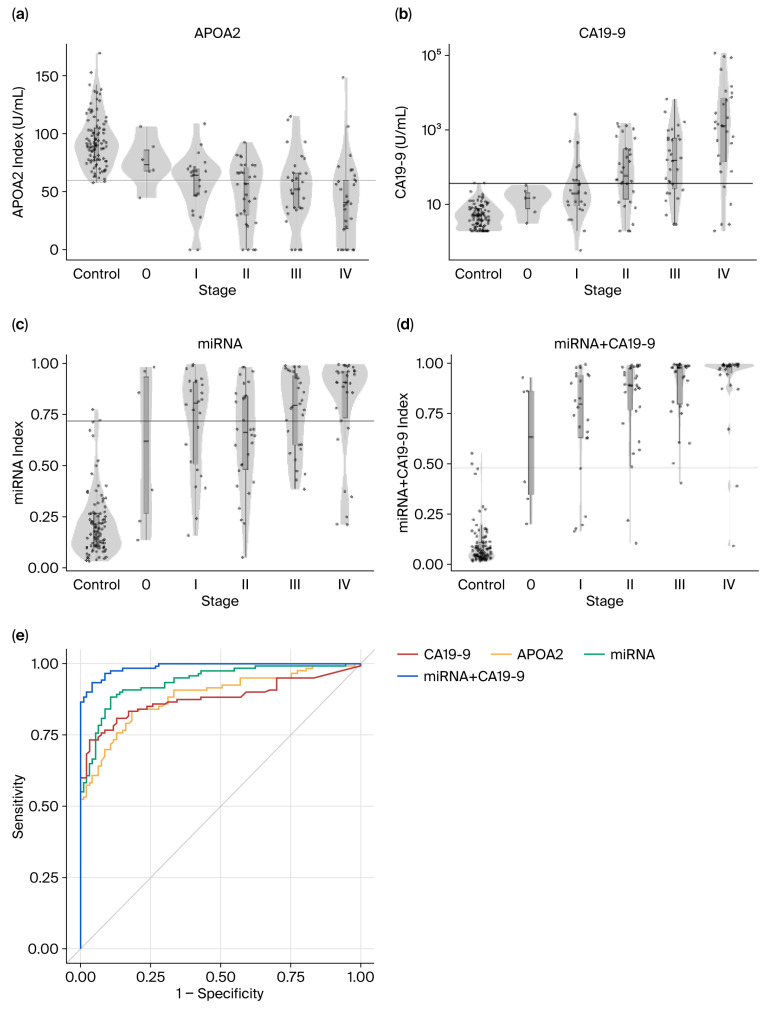

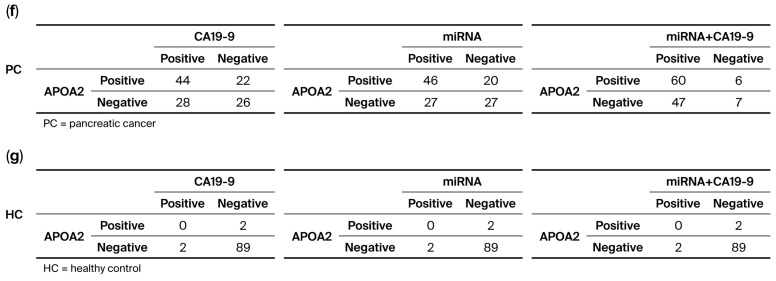
The miRNA and miRNA+CA19-9 models show better distinction performance than APOA2 and CA19-9 for pancreatic cancer patients at all stages. (**a**–**d**) Box plots of APOA2, CA19-9, the miRNA model, and the miRNA+CA19-9 model of healthy participants and pancreatic cancer patients at each stage (0, I, II, III, and IV). (**e**) ROC curve for the performance of APOA2 (orange), CA19-9 alone (red), the miRNA model (green), and the miRNA+CA19-9 model (blue). (**f**,**g**) Comparison of the distinction between APOA2 vs. CA19-9, APOA2 vs. the miRNA model, and APOA2 vs. the miRNA+CA19-9 model.

The diagnostic performance of serum APOA2, CA19-9, the miRNA model, and the combined miRNA+CA19-9 model was assessed. Figure 1a–d present APOA2 index levels, CA19-9 concentrations, miRNA index levels, and miRNA+CA19-9 index levels in healthy controls and pancreatic cancer patients stratified by stage. With advancing disease stage, APOA2 index levels declined, whereas CA19-9, miRNA, and miRNA+CA19-9 index levels increased. Relative to APOA2 and CA19-9, both the miRNA model and the miRNA+CA19-9 model demonstrated superior discriminative ability.

Figure 1e shows ROC curves for the four biomarkers/models, and ROC analysis likewise indicated better discrimination with the miRNA-based models than with APOA2 or CA19-9. AUC, specificity, sensitivity, and stage-specific sensitivities are summarized in Table 2. When pancreatic cancer cases were compared with healthy controls, the AUCs were 0.89 (95% CI, 0.84–0.93) for APOA2, 0.88 (95% CI, 0.84–0.93) for CA19-9, 0.94 (95% CI, 0.91–0.97) for the miRNA model, and 0.99 (95% CI, 0.98–1.00) for the miRNA+CA19-9 model (Table 2). Discriminative performance comparisons of APOA2 vs. CA19-9, APOA2 vs. the miRNA model, and APOA2 vs. the miRNA+CA19-9 model in pancreatic cancer patients and healthy controls are shown in Figure 1f–g. In pancreatic cancer patients, 39% (47/120) were APOA2-negative but miRNA+CA19-9-positive.

Overall, these findings suggest that the miRNA and miRNA+CA19-9 models outperform APOA2 and CA19-9 in distinguishing pancreatic cancer from healthy controls across all disease stages.

### 3.3. Correlation Between APOA2 and the miRNA Model, and Between APOA2 and the miRNA+CA19-9 Model

**Figure 2:**
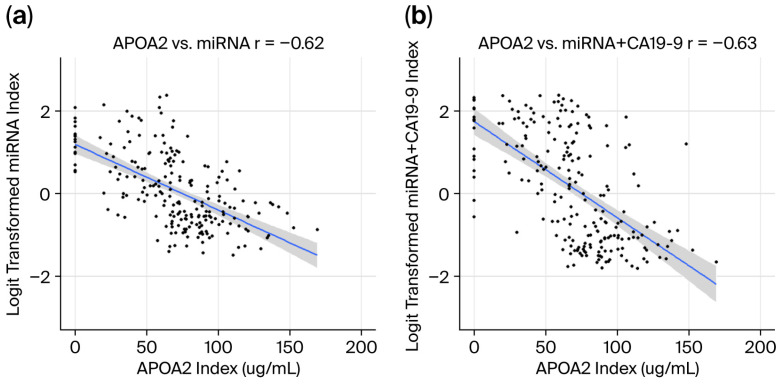
Negative correlations between APOA2 and miRNA, as well as between APOA2 and the miRNA+CA19-9 model. (**a**) Correlation between the APOA2 index and the miRNA model index. (**b**) Correlation between the APOA2 index and the miRNA+CA19-9 model index. The miRNA model index and the miRNA+CA19-9 model index were logit-transformed. An r value of 0.65 indicates a meaningful positive correlation. Strong negative correlations were observed between APOA2 and miRNA and between APOA2 and the miRNA+CA19-9 model.

The correlations between the APOA2 index and the miRNA model index and between the APOA2 index and the miRNA+CA19-9 model index are shown in Figure 2. The Spearman correlation coefficient between the APOA2 index and the miRNA model index was 0.62, and that between the APOA2 index and the miRNA+CA19-9 model index was 0.63. These results indicate that there are negative correlations between APOA2 and miRNA, as well as between APOA2 and the miRNA+CA19-9 model.

### 3.4. Distinction Performance for Early Stage Pancreatic Cancer Patients

**Figure 3:**
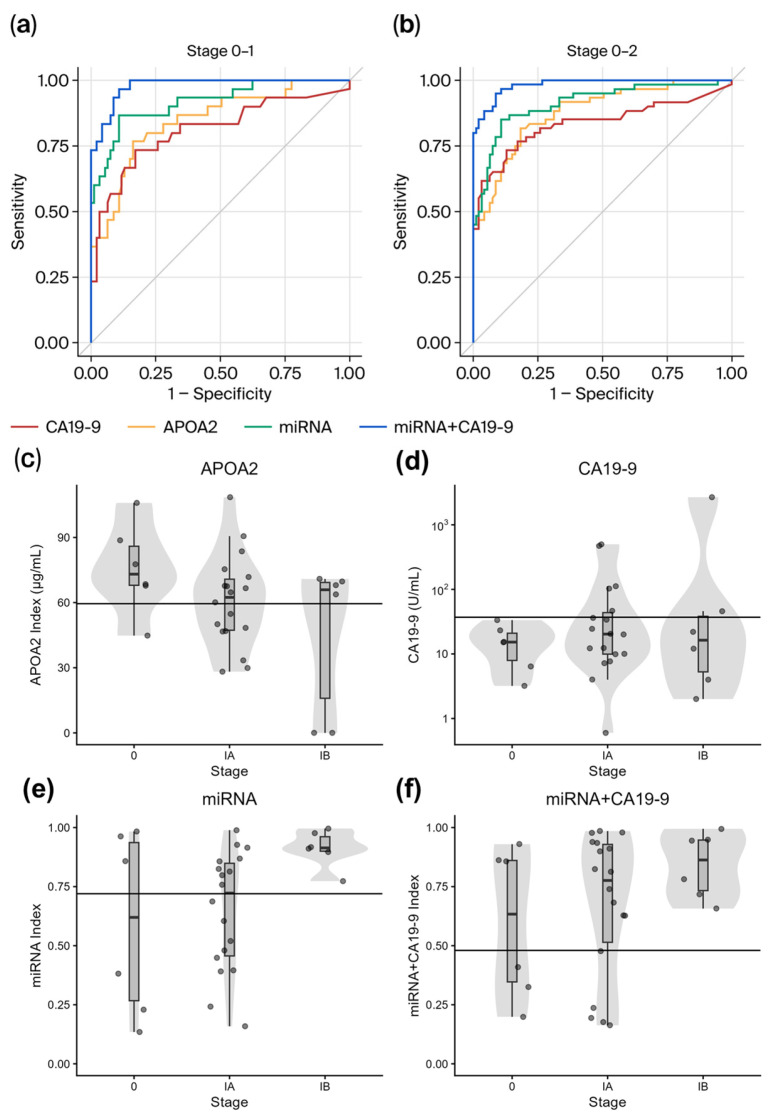
The miRNA and miRNA+CA19-9 models show better distinction performance than APOA2 for early-stage pancreatic cancer patients. (**a**,**b**) ROC curve for the performance of APOA2 (orange), CA19-9 alone (red), the miRNA model (green), and the miRNA+CA19-9 model (blue) at stage 0-I (**a**) and stage 0-II (**b**). The miRNA and miRNA+CA19-9 models show better performance than APOA2. (**c**–**f**) Box plots of APOA2, CA19-9, the miRNA model, and the miRNA+CA19-9 model of healthy participants and pancreatic cancer patients at each stage (0, IA, and IB).

Figure 3a,b present ROC curves for APOA2, CA19-9, the miRNA model, and the combined miRNA+CA19-9 model in patients with stage 0–I and stage 0–II pancreatic cancer. The AUCs, specificities, sensitivities, and stage-specific sensitivities for stages 0, I, and II are summarized in Table 3 and Table 4 for each marker/model. In discriminating stage 0–I and stage 0–II pancreatic cancer, the miRNA and miRNA+CA19-9 models performed better than APOA2. Additionally, we stratified stage I into IA and IB (Figure 3c–f). Notably, the miRNA+CA19-9 model demonstrated superior distinction over APOA2 even in patients with Stage 0–IA, where imaging-based detection is often challenging. The false negative rate of APOA2, CA19-9, miRNA model, and miRNA+CA19-9 model in patients with Stage 0 were 83% (5/6), 100% (6/6), 50% (6/6), and 50% (6/6). The false negative rate with Stage IA were 56% (10/18), 72% (13/18), 50% (9/18), and 28% (5/18). The false negative rate with Stage IB were 67% (4/6), 67% (4/6), 0% (0/6), and 0% (0/6). These results indicate that the miRNA and miRNA+CA19-9 models showed better distinction performance than APOA2 for early-stage pancreatic cancer patients.

### 3.5. Distinction Performance of CA19-9, the miRNA Model, and the miRNA+CA19-9 Model for APOA2-Negative Patient Samples

**Figure 4:**
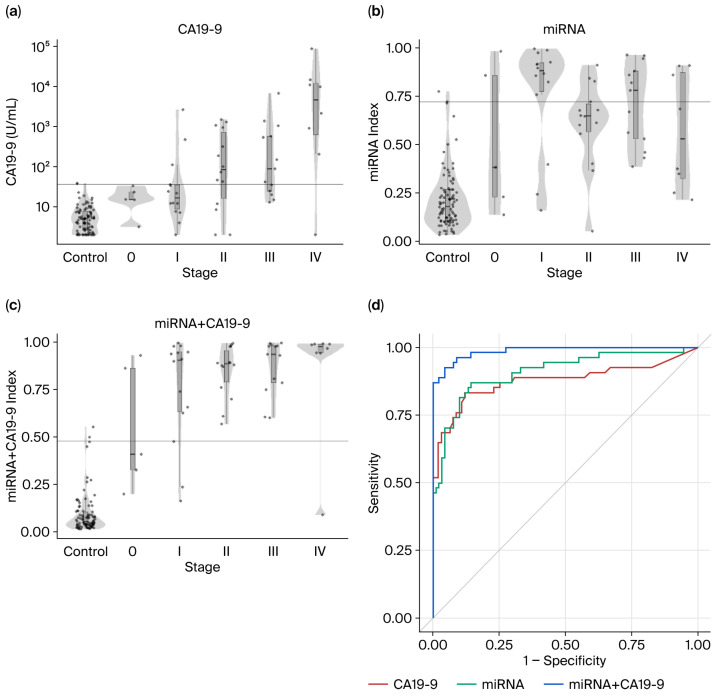
ROC curve for the performance of CA19-9 alone, the miRNA model, and the miRNA+CA19-9 model in APOA2-negative samples. (**a**–**c**) Box plots of CA19-9 and indices of the miRNA and miRNA+CA19-9 models of APOA2-negative healthy participants and pancreatic cancer patients at each stage (0, I, II, III, and IV). (**d**) ROC curve for the performance of CA19-9 alone (red), the miRNA model (green), and the miRNA+CA19-9 model (blue) in APOA2-negative samples.

Samples classified as APOA2-negative (APOA2 index > 59.5 µg/mL; pancreatic cancer: 54/120; healthy controls: 91/93) were extracted and further evaluated using CA19-9, the miRNA model, and the combined miRNA+CA19-9 model. Figure 4a–c shows CA19-9 values, miRNA model index values, and miRNA+CA19-9 model index values in healthy controls and pancreatic cancer patients by stage. ROC curves for CA19-9, the miRNA model, and the miRNA+CA19-9 model in the APOA2-negative subset are presented in Figure 4d. Performance metrics—including AUC, specificity, sensitivity, and stage-specific sensitivities—are summarized in Table 5 for these three approaches within APOA2-negative samples. The miRNA+CA19-9 model achieved an AUC of 0.99 and yielded a positive rate of 87% (47/54).

## 4. Discussion

In this study, we compared the diagnostic performances of the miRNA, miRNA+CA19-9, and APOA2 models for pancreatic cancer. The miRNA and miRNA+CA19-9 models demonstrated a superior ability to distinguish patients with pancreatic cancer from non-cancer individuals compared with APOA2, particularly in terms of identifying patients with stage 0–I and 0–II pancreatic cancers. Interestingly, strong negative correlations were observed between APOA2 and the miRNA model, as well as between APOA2 and the miRNA+CA19-9 model. APOA2 reflects the change in the micro-environment caused by a pancreatic disorder [15], and the strong correlation between APOA2 and miRNA may indicate that miRNA also reflects the change in the micro-environment.

miRNAs are short non-coding RNAs approximately 18–24 nucleotides long [17,18]. They regulate gene expression by promoting degradation of target mRNAs or by inhibiting translation. Because circulating miRNAs are stable even in challenging biofluids such as serum and pancreatic juice, they have attracted attention as liquid biopsy biomarkers. This durability is believed to arise in part because miRNAs are packaged within extracellular vesicles and/or associated with the RNA-induced silencing complex (RISC) via the Ago2 (Argonaute2) protein, which shields them from RNase-driven breakdown [19,20,21]. Multiple retrospective studies have shown that the levels of certain plasma or serum miRNAs can differentiate pancreatic cancer patients from healthy individuals and from those with benign pancreatic disorders [16,22,23,24,25,26,27,28,29,30,31,32,33]. In this study, we developed a diagnostic model using next-generation sequencing (NGS) and automated machine learning (AutoML) based on 100 abundantly expressed serum miRNAs together with CA19-9 [16].

Apolipoprotein A2 (APOapoA2) is a major apolipoprotein component of high-density lipoprotein, and it circulates as a disulfide-linked homodimer with distinct C-terminal processing patterns (APOapoA2 isoforms) [34]. Previous studies have shown that in chronic pancreatitis, intraductal papillary mucinous neoplasms (IPMNs), and pancreatic cancer, abnormalities in pancreatic exocrine function result in altered exopeptidase activity, the enzymes responsible for cleaving the C-terminal end of APOapoA2. Consequently, the serum concentration of APOApoA2-ATQ/AT, one of the apoA2 isoforms, is decreased compared with that in healthy individuals [15,35,36]. ELISA kits have been developed to quantify APOapoA2 isoforms for the early detection of pancreatic cancer and high-risk individuals [15].

In the present study, the miRNA and miRNA+CA19-9 models maintained high ROC performance, even in pancreatic cancer patients who tested negative with the APOA2 model. This finding suggests that alterations in circulating miRNAs occur at a high frequency, even in patients with pancreatic cancer who do not exhibit abnormalities in pancreatic exocrine function. Conversely, the strong negative correlations observed between the miRNA-based models and the APOA2 model indicate that these two approaches may function in a complementary manner.

It is important to note that this study has several limitations. First, almost all participants were Japanese, and all samples were collected at medical institutions within a single country; therefore, it is uncertain whether these results can be generalized to other ethnic groups. Second, this retrospective study only included patients with pancreatic cancer and healthy participants. Because individuals with benign pancreatic diseases such as chronic pancreatitis or IPMN were not included as controls, the diagnostic performance of these models in more clinically challenging, real-world differential diagnostic settings remains unknown. Third, the number of patients with stage 0 and stage I pancreatic cancers was relatively small; thus, stage-specific sensitivities should be interpreted with caution. Fourth, potential confounding by age exists because the healthy volunteer blood donors were younger than the patients with pancreatic cancer. Fifth, it remains unclear whether this miRNA model also detects other types of cancers, such as biliary or colorectal cancers. Sixth, the biological origins of the 100 serum miRNAs and their functional roles in pancreatic cancer remain unknown. Seventh, the cost is one of the limitations of this study. In our method, all 100 miRNAs are measured simultaneously in a single next-generation sequencing (NGS) run, not individually. In addition, NGS allows multiple samples to be multiplexed in a single run, which further reduces the per-sample cost. Furthermore, miRNA sequencing targets a limited region of the transcriptome compared to conventional RNA-Seq, making it inherently more cost-effective. We believe our method has the potential to serve as a cost-feasible screening tool. Eighth, diabetes mellitus is a known risk factor for pancreatic cancer [37,38,39]. However, the proportion of patients with type 2 diabetes mellitus was significantly higher in the PC group than in the HC group, which may represent a potential source of bias. On the other hand, it has been reported that pancreatic ductal adenocarcinoma can induce peripheral insulin resistance [40]. Therefore, the significantly higher prevalence of type 2 diabetes mellitus in the PC group compared with the HC group may reflect impaired glucose tolerance associated with PDAC rather than a pre-existing risk factor alone. Eighth, although a comprehensive model integrating miRNA, CA19-9, and APOA2 may further improve diagnostic performance, the present study used only the validation cohort from a prior study and was not designed for new model development. Future studies with a dedicated discovery–validation cohort design are warranted to develop such an integrated model.

## 5. Conclusions

In conclusion, the miRNA and miRNA+CA19-9 models showed better distinction performance compared with APOA2 for pancreatic cancer patients of all stages, particularly in terms of discriminating between patients with stages 0–I and 0–II pancreatic cancer. Strong negative correlations were observed between APOA2 and the miRNA model, as well as between APOA2 and the miRNA+CA19-9 model.

## Figures and Tables

**Table 1:** Characteristics of pancreatic cancer patients and healthy controls.

	PC	HC	*p*-Value (PC vs. HC)
Total [no.]	120	93	
Sex			
Male [no.]	73	45	0.070 ^b^
Female [no.]	47	48	
Age			
Median (range) [y]	72.5 (38–86)	60 (40–83)	<0.001 ^a^
History of smoking			
Current [no.]	17	10	0.161 ^b^
Former [no.]	53	32	
Never [no.]	50	51	
Drinking habits			
Everyday [no.]	33	18	0.067 ^b^
Sometimes [no.]	26	35	
Non [no.]	60	40	
NA [no.]	1	0	
Diabetes mellitus			
No [no.]	73	79	<0.001 ^b^
Yes [no.]	47	14	
CA19-9			
Median (range) [U/mL]	44.7 (0.6–1.2 × 10^5^)	4.7 (2–38.1)	0.007 ^a^
Stage [no.]			
0	6		
I	24		
II	30		
III	30		
IV	30		

PC—pancreatic cancer; HC—healthy control; NA—not available. ^a^ Student’s *t*-test. ^b^ Pearson’s chi-squared test. The *p*-value is for pancreatic cancers (PCs) vs. healthy controls (HCs). There was a significant difference in age, history of smoking, and diabetes mellitus between patients with pancreatic cancer and healthy participants.

**Table 2:** Performance of APOA2, CA19-9, the miRNA model, and the miRNA+CA19-9 model in determining pancreatic cancer patients from healthy participants.

	APOA2	CA19-9	miRNA Model	miRNA+CA19-9 Model
AUC	0.89	0.88	0.94	0.99
95% CI	0.84–0.93	0.83–0.93	0.91–0.97	0.98–1.0
Specificity	0.98 ^a^	0.98 ^b^	0.98 ^c^	0.98 ^d^
95% CI	0.95–1.0	0.95–1.0	0.95–1.0	0.95–1.0
Sensitivity	0.55	0.61	0.61	0.89
95% CI	0.46–0.63	0.52–0.70	0.53–0.70	0.83–0.94
Each Stage Sensitivity				
Stage 0	0.17	0	0.5	0.5
	0.00–0.50	0.00–0.00	0.17–0.83	0.17–0.83
Stage I	0.42	0.29	0.63	0.79
	0.25–0.63	0.13–0.50	0.42–0.79	0.63–0.96
Stage II	0.53	0.67	0.4	0.93
	0.37–0.70	0.50–0.83	0.23–0.57	0.83–1.00
Stage III	0.57	0.7	0.67	0.97
	0.40–0.73	0.53–0.87	0.50–0.83	0.90–1.00
Stage IV	0.73	0.83	0.77	0.93
	0.57–0.90	0.70–0.97	0.60–0.90	0.83–1.00

^a^ Threshold = 59.5 µg/mL; ^b^ threshold = 37 U/mL; ^c^ threshold = 0.72; ^d^ threshold = 0.48.

**Table 3:** Performance of APOA2, CA19-9, the miRNA model, and the miRNA+CA19-9 model in discriminating stage 0–I pancreatic cancer patients from healthy participants.

	APOA2	CA19-9	miRNA Model	miRNA+CA19-9 Model
AUC	0.85	0.81	0.92	0.98
95% CI	0.760.93	0.71–0.92	0.86–0.98	0.96–1.00
Specificity	0.98	0.98	0.98	0.98
95% CI	0.95–1.00	0.95–1.00	0.95–1.00	0.95–1.00
Sensitivity	0.37	0.32	0.6	0.73
95% CI	0.20–0.53	0.10–0.40	0.43–0.77	0.57–0.87
Each Stage Sensitivity				
Stage 0	0.17	0	0.5	0.5
	0.00–0.50	0.00–0.00	0.17–0.83	0.17–0.83
Stage I	0.42	0.29	0.63	0.79
	0.25–0.63	0.13–0.50	0.42–0.79	0.63–0.96

**Table 4:** Performance of APOA2, CA19-9, the miRNA model, and the miRNA+CA19-9 model in discriminating stage 0–II pancreatic cancer patients from healthy participants.

	APOA2	CA19-9	miRNA Model	miRNA+CA19-9 Model
AUC	0.88	0.83	0.91	0.98
95% CI	0.82–0.93	0.76–0.91	0.86–0.96	0.97–1.00
Specificity	0.98	0.98	0.98	0.98
95% CI	0.95–1.00	0.95–1.00	0.95–1.00	0.95–1.00
Sensitivity	0.45	0.45	0.5	0.83
95% CI	0.33–0.58	0.33–0.57	0.37–0.62	0.73–0.92
Each Stage Sensitivity				
Stage 0	0.17	0	0.5	0.5
	0.00–0.50	0.00–0.00	0.17–0.83	0.17–0.83
Stage I	0.42	0.29	0.63	0.79
	0.25–0.63	0.13–0.50	0.42–0.79	0.63–0.96
Stage II	0.53	0.67	0.4	0.93
	0.37–0.70	0.50–0.83	0.23–0.57	0.83–1.00

**Table 5:** Performance of CA19-9, the miRNA model, and the miRNA+CA19-9 model in discriminating APOA2-negative pancreatic cancer patients from APOA2-negative healthy participants.

	CA19-9	miRNA Model	miRNA+CA19-9 Model
AUC	0.88	0.91	0.99
95% CI	0.81–0.95	0.86–0.96	0.97–1.00
Specificity	0.98	0.98	0.98
95% CI	0.95–1.00	0.95–1.00	0.95–1.00
Sensitivity	0.52	0.5	0.87
95% CI	0.39–0.65	0.37–0.63	0.78–0.94
Each Stage Sensitivity			
Stage 0	0	0.4	0.4
	0.00–0.00	0.00–0.80	0.00–0.80
Stage I	0.21	0.78	0.78
	0.00–0.43	0.57–1.00	0.57–1.00
Stage II	0.71	0.29	1
	0.43–0.93	0.07–0.50	1.00–1.00
Stage III	0.62	0.54	1
	0.39–0.84	0.23–0.77	1.00–1.00
Stage IV	0.88	0.38	0.88
	0.63–1.00	0.13–0.75	0.63–1.00

## Data Availability

The main data supporting the results of this study are available in this paper. The raw and analyzed datasets generated during this study are available for research purposes from the corresponding authors upon reasonable request. As they contain personal and patient data, they are available for research purposes pending the completion of adequate paperwork, the protection of personal data, and ethical approval.

## Supplementary Materials

The following supporting information can be downloaded at: https://www.mdpi.com/article/10.3390/cancers18071177/s1, Table S1. The 100 miRNAs and their sequences used for miRNA and miRNA+CA19-9 model constructions.

## Abbreviations

The following abbreviations are used in this manuscript:

APOA2	Apolipoprotein A2
APOA2-ATQ/AT	Apolipoprotein A2 Isoform ATQ/AT
AutoML	Automated Machine Learning
CA19-9	Carbohydrate Antigen 19-9
ELISA	Enzyme-Linked Immunosorbent Assay
FGFR2	Fibroblast Growth Factor Receptor 2
HRI	High-Risk Individual
IPMN	Intraductal Papillary Mucinous Neoplasm
miRNA	MicroRNA
NGS	Next-Generation Sequencing
PDAC	Pancreatic Ductal Adenocarcinoma
