# Supplementary material for: Comparison of Comprehensive Serum miRNA Sequencing and Apolipoprotein A2 Isoforms for Early Detection of Pancreatic Cancer
Source: Cancers (Basel). 2026 Apr 7;18(7):1177. doi: 10.3390/cancers18071177 (PMC13072215; doi:10.3390/cancers18071177)
Supplement: Supplementary file 1 [file cancers-18-01177-s001.zip › cancers-4064902-supplementary.pdf]

Supplementary Materials: TableS1. The 100 miRNAs and their sequences used for miRNA and miRNA+CA19-9 model constructions.

Number	miRNA	Sequence
1	hsa-let-7a-5p	UGAGGUAGUAGGUUGUAUAGUU
2	hsa-let-7b-5p	UGAGGUAGUAGGUUGUGUGGUU
3	hsa-let-7c-5p	UGAGGUAGUAGGUUGUAUGGUU
4	hsa-let-7d-3p	CUAUACGACCUGCUGCCUUUCU
5	hsa-let-7d-5p	AGAGGUAGUAGGUUGCAUAGUU
6	hsa-let-7e-5p	UGAGGUAGGAGGUUGUAUAGUU
7	hsa-let-7f-5p	UGAGGUAGUAGAUUGUAUAGUU
8	hsa-let-7g-5p	UGAGGUAGUAGUUUGUACAGUU
9	hsa-let-7i-5p	UGAGGUAGUAGUUUGUGCUGUU
10	hsa-miR-101-3p	UACAGUACUGUGAUAACUGAA
11	hsa-miR-103a-3p	AGCAGCAUUGUACAGGGCUAUGA
12	hsa-miR-107	AGCAGCAUUGUACAGGGCUAUCA
13	hsa-miR-10a-5p	UACCCUGUAGAUCCGAAUUUGUG
14	hsa-miR-10b-5p	UACCCUGUAGAACCGAAUUUGUG
15	hsa-miR-122-5p	UGGAGUGUGACAAUGGUGUUUG
16	hsa-miR-125a-5p	UCCCUGAGACCCUUUAACCUGUGA
17	hsa-miR-125b-5p	UCCCUGAGACCCUAACUUGUGA
18	hsa-miR-126-3p	UCGUACCGUGAGUAAUAAUGCG
19	hsa-miR-126-5p	CAUUAUUACUUUUGGUACGCG
20	hsa-miR-1277-5p	AAAUAUAUAUAUAUAUGUACGUAU
21	hsa-miR-128-3p	UCACAGUGAACCGGUCUCUUU
22	hsa-miR-130a-3p	CAGUGCAAUGUUAAAAGGGCAU
23	hsa-miR-140-3p	UACCACAGGGUAGAACCACGG
24	hsa-miR-142-3p	UGUAGUGUUUCCUACUUUAUGGA
25	hsa-miR-142-5p	CAUAAAGUAGAAAGCACUACU
26	hsa-miR-143-3p	UGAGAUGAAGCACUGUAGCUC
27	hsa-miR-144-3p	UACAGUAUAGAUGAUGUACU
28	hsa-miR-144-5p	GGAUAUCAUCAUAUACUGUAAG
29	hsa-miR-146a-5p	UGAGAACUGAAUUCCAUGGGUU
30	hsa-miR-146b-5p	UGAGAACUGAAUUCCAUAGGCU
31	hsa-miR-148a-3p	UCAGUGCACUACAGAACUUUGU
32	hsa-miR-148b-3p	UCAGUGCAUCACAGAACUUUGU
33	hsa-miR-150-5p	UCUCCCAACCCUUGUACCAGUG

34	hsa-miR-151a-3p	CUAGACUGAAGCUCCUUGAGG
35	hsa-miR-152-3p	UCAGUGCAUGACAGAACUUGG
36	hsa-miR-155-5p	UUAAUGCUAAUCGUGAUAGGGGU
37	hsa-miR-15a-5p	UAGCAGCACAUAAUGGUUUGUG
38	hsa-miR-15b-5p	UAGCAGCACAUCAUGGUUUACA
39	hsa-miR-16-5p	UAGCAGCACGUAAAUAUUGGCG
40	hsa-miR-181a-5p	AACAUUCAACGCUGUCGGUGAGU
41	hsa-miR-182-5p	UUUGGCAAUGGUAGAACUCACACU
42	hsa-miR-185-5p	UGGAGAGAAAGGCAGUUCCUGA
43	hsa-miR-186-5p	CAAAGAAUUCUCCUUUUGGGCU
44	hsa-miR-190a-5p	UGAUAUGUUUGAUAUAUUAGGU
45	hsa-miR-191-5p	CAACGGAAUCCCAAAAGCAGCUG
46	hsa-miR-192-5p	CUGACCUAUGAAUUGACAGCC
47	hsa-miR-194-5p	UGUAACAGCAACUCCAUGUGGA
48	hsa-miR-199a-3p	ACAGUAGUCUGCACAUUGGUUA
49	hsa-miR-199b-3p	ACAGUAGUCUGCACAUUGGUUA
50	hsa-miR-19a-3p	UGUGCAAAUCUAUGCAAAACUGA
51	hsa-miR-19b-3p	UGUGCAAAUCCAUGCAAAACUGA
52	hsa-miR-20a-5p	UAAAGUGCUUAUAGUGCAGGUAG
53	hsa-miR-20b-5p	CAAAGUGCUCAUAGUGCAGGUAG
54	hsa-miR-21-5p	UAGCUUAUCAGACUGAUGUUGA
55	hsa-miR-221-3p	AGCUACAUUGUCUGCUGGGUUUC
56	hsa-miR-222-3p	AGCUACAUCUGGCUACUGGGU
57	hsa-miR-223-3p	UGUCAGUUUGUCAAAUACCCCA
58	hsa-miR-22-3p	AAGCUGCCAGUUGAAGAACUGU
59	hsa-miR-23a-3p	AUCACAUUGCCAGGGAUUUCC
60	hsa-miR-23b-3p	AUCACAUUGCCAGGGAUUACC
61	hsa-miR-24-3p	UGGCUCAGUUCAGCAGGAACAG
62	hsa-miR-25-3p	CAUUGCACUUGUCUCGGUCUGA
63	hsa-miR-26a-5p	UUCAAGUAAUCCAGGAUAGGCU
64	hsa-miR-26b-5p	UUCAAGUAAUUCAGGAUAGGU
65	hsa-miR-27a-3p	UUCACAGUGGCUAAGUUCCGC
66	hsa-miR-27b-3p	UUCACAGUGGCUAAGUUCUGC
67	hsa-miR-28-3p	CACUAGAUUGUGAGCUCCUGGA
68	hsa-miR-29a-3p	UAGCACCAUCUGAAAUCGGUUA
69	hsa-miR-29b-3p	UAGCACCAUUUGAAAUCAGUGUU
70	hsa-miR-29c-3p	UAGCACCAUUUGAAAUCGGUUA
71	hsa-miR-30a-5p	UGUAAACAUCCUCGACUGGAAG
72	hsa-miR-30c-5p	UGUAAACAUCCUACACUCUCAGC

73	hsa-miR-30d-5p	UGUAAACAUCCCCGACUGGAAG
74	hsa-miR-30e-5p	UGUAAACAUCCUUGACUGGAAG
75	hsa-miR-320a	AAAAGCUGGGUUGAGAGGGCGA
76	hsa-miR-32-5p	UAUUGCACAUUACUAAGUUGCA
77	hsa-miR-335-5p	UCAAGAGCAAUAACGAAAAAUGU
78	hsa-miR-338-3p	UCCAGCAUCAGUGAUUUUGUUG
79	hsa-miR-340-5p	UUAUAAAGCAAUGAGACUGAUU
80	hsa-miR-342-3p	UCUCACACAGAAAUCGCACCCGU
81	hsa-miR-3613-5p	UGUUGUACUUUUUUUUUUGUUC
82	hsa-miR-361-5p	UUAUCAGAAUCUCCAGGGGUAC
83	hsa-miR-363-3p	AAUUGCACGGUAUCCAUCUGUA
84	hsa-miR-374a-5p	UUAUAAUACAACCUGAUAAGUG
85	hsa-miR-423-5p	UGAGGGGCAGAGAGCGAGACUUU
86	hsa-miR-424-5p	CAGCAGCAAUUCAUGUUUUGAA
87	hsa-miR-425-5p	AAUGACACGAUCACUCCCGUUGA
88	hsa-miR-451a	AAACCGUUACCAUUACUGAGUU
89	hsa-miR-454-3p	UAGUGCAAUAUUGCUUAUAGGGU
90	hsa-miR-484	UCAGGCUCAGUCCCCUCCCGAU
91	hsa-miR-486-3p	CGGGGCAGCUCAGUACAGGAU
92	hsa-miR-486-5p	UCCUGUACUGAGCUGCCCCGAG
93	hsa-miR-584-5p	UUAUGGUUUGCCUGGGACUGAG
94	hsa-miR-629-5p	UGGGUUUACGUUGGGAGAACU
95	hsa-miR-660-5p	UACCCAUUGCAUAUCGGAGUUG
96	hsa-miR-744-5p	UGCGGGGCUAGGGCUAACAGCA
97	hsa-miR-7-5p	UGGAAGACUAGUGAUUUUGUUGU
98	hsa-miR-92a-3p	UAUUGCACUUGUCCCGGCCUGU
99	hsa-miR-93-5p	CAAAGUGCUGUUCGUGCAGGUAG
100	hsa-miR-98-5p	UGAGGUAGUAAGUUGUAUUGUU
